# A new scheme for strain typing of methicillin-resistant *Staphylococcus aureus* on the basis of matrix-assisted laser desorption ionization time-of-flight mass spectrometry by using machine learning approach

**DOI:** 10.1371/journal.pone.0194289

**Published:** 2018-03-13

**Authors:** Hsin-Yao Wang, Tzong-Yi Lee, Yi-Ju Tseng, Tsui-Ping Liu, Kai-Yao Huang, Yung-Ta Chang, Chun-Hsien Chen, Jang-Jih Lu

**Affiliations:** 1 Department of Laboratory Medicine, Chang Gung Memorial Hospital at Linkou, Taoyuan City, Taiwan; 2 Department of Computer Science & Engineering, Yuan Ze University, Taoyuan City, Taiwan; 3 School of Science and Engineering, The Chinese University of Hong Kong, Shenzhen, China; 4 Department of Information Management, Chang Gung University, Taoyuan City, Taiwan; 5 Department of Medical Biotechnology and Laboratory Science, Chang Gung University, Taoyuan City, Taiwan; Universitatsklinikum Munster, GERMANY

## Abstract

Methicillin-resistant *Staphylococcus aureus* (MRSA), one of the most important clinical pathogens, conducts an increasing number of morbidity and mortality in the world. Rapid and accurate strain typing of bacteria would facilitate epidemiological investigation and infection control in near real time. Matrix-assisted laser desorption ionization-time of flight (MALDI-TOF) mass spectrometry is a rapid and cost-effective tool for presumptive strain typing. To develop robust method for strain typing based on MALDI-TOF spectrum, machine learning (ML) is a promising algorithm for the construction of predictive model. In this study, a strategy of building templates of specific types was used to facilitate generating predictive models of methicillin-resistant *Staphylococcus aureus* (MRSA) strain typing through various ML methods. The strain types of the isolates were determined through multilocus sequence typing (MLST). The area under the receiver operating characteristic curve (AUC) and the predictive accuracy of the models were compared. ST5, ST59, and ST239 were the major MLST types, and ST45 was the minor type. For binary classification, the AUC values of various ML methods ranged from 0.76 to 0.99 for ST5, ST59, and ST239 types. In multiclass classification, the predictive accuracy of all generated models was more than 0.83. This study has demonstrated that ML methods can serve as a cost-effective and promising tool that provides preliminary strain typing information about major MRSA lineages on the basis of MALDI-TOF spectra.

## Introduction

*Staphylococcus aureus* is one of the most important clinical pathogens that results in various types of infection. Particularly, methicillin-resistant *Staphylococcus aureus* (MRSA) has become a serious issue worldwide, because it is associated with increased morbidity and mortality [1–5]. Epidemiologically, early detection of a possible MRSA outbreak and appropriate infection control would limit its spread. For epidemiological investigation and surveillance, strain typing of MRSA in real time is essential [3, 5, 6]. Several molecular methods, including pulsed-field gel electrophoresis (PFGE), *S*. *aureus* protein A (spa) typing, and multilocus sequence typing (MLST), have been developed for strain typing of MRSA [3, 6, 7]. These DNA-based analyses provide accurate and detailed subspecies information. However, these methods are labor-intensive, time-consuming, and cost-ineffective [3, 5, 7, 8]. For instance, several days are necessary for strain typing through PFGE. Thus, their wide application in clinical practice is restricted because the requirements of rapidity and cost-effectiveness for strain typing are not satisfied.

Protein expression pattern obtained from matrix-assisted laser desorption ionization-time of flight (MALDI-TOF) mass spectrometry is an alternative tool for timely, cost-effective, and reliable strain typing. In recent years, MALDI-TOF spectral analysis has been widely used in clinical microbiology for the identification of bacteria, fungi, and mycobacteria [3, 9–12]. The distinctive features of MALDI-TOF spectral analysis include rapidity, cost-effectiveness, and accuracy. Only several minutes are required to correctly identify an isolate by using MALDI-TOF spectral analysis. It is also more cost-effective than PFGE, spa typing, and MLST. Moreover, it produces reliable discrimination results at the species level, with high reproducibility [9–11]. Because of the aforementioned characteristics, many clinical microbiology laboratories routinely utilize MALDI-TOF for bacterial identification. In addition to the identification of bacterial species, some researchers have utilized MALDI-TOF spectra for subspecies discrimination (e.g., strain typing) [3, 6, 7, 9, 11, 13, 14].

Recently, several methods have been developed to analyze MALDI-TOF spectra for subspecies discrimination. Some studies have conducted visual examination of the MALDI-TOF pseudo-gel or spectra to discover strain-specific peaks [3, 13, 15]. Other studies have identified strain-representative peaks on MALDI-TOF spectra through software [1, 7, 16]. In these studies, the presence or absence of specific peaks was considered the classification rules for subspecies discrimination [3, 5, 15]. However, to date, inconsistent results have been obtained. The underlying reasons are complex. First, the MALDI-TOF spectra of highly related strain are similar. The subtle difference among the spectra would be difficult to interpret correctly by visual examination [6, 7, 17]. Moreover, protein expression differences among strains may be also present in the non-ribosomal proteins [9, 13]. Not only the presence or absence of proteins but also their expression levels are considered in the discrimination of microbial subspecies at the strain level [5, 6]. To resolve the issues, machine learning (ML) methods may generate more detailed information.

ML methods are multivariate analysis algorithms. ML models can predict unknown cases by learning the pattern of multivariate data in training cases. The decision tree (DT) induction algorithm, support vector machine (SVM), and k-nearest neighbor (KNN) are robust and widely applied ML methods for real-world discrimination problems [18–21]. This study aims to develop a framework of applying machine learning method to the analysis of MALDI-TOF spectra. The MRSA was used as a case study for demonstrating the performance of the proposed methodology. To successfully applying ML methods in strain typing, features obtained from MALDI-TOF spectra should be well-defined. In this study, method of building strain-specific template was developed to cope with the issue of “peak shift” [22] and defining features. The MALDI-TOF spectra of clinically obtained MRSA isolates were used to train models based on DT, SVM, and KNN for the discrimination of major MRSA lineages. The performance levels of the models for strain typing of MRSA were evaluated and compared.

## Material and methods

### Bacterial isolates

A total of 125 clinical MRSA isolates were collected from 2009 to 2014 at the Linkou branch of Chang Gung Memorial Hospital (CGMH), Taiwan. The isolates were all recovered from inpatients’ blood specimens. The blood specimens were obtained from department of laboratory medicine of CGMH, which received all the specimens throughout the various wards. The clinical blood samples were cultured in trypticase soy broth (Becton Dickinson, MD, USA). Positive growth of bacteria was detected by the automated detection system (BD BACTEC^™^ FX; Becton Dickinson, MD, USA). Subculture from the positive blood bottle onto blood plate agar (Becton Dickinson, MD, USA) was performed. *S*. *aureus* was identified on the basis of colony morphology, microscopic examination, coagulase test, catalase test, and MALDI-TOF spectra. According to Clinical & Laboratory Standards Institute (CLSI) guidelines, we used cefoxitin disk as the surrogate agent to determine resistance to oxacillin. The isolates were frozen at −70°C until use. The susceptibility test was repeated after re-cultivating the isolates from storage. Moreover, we have also performed *mecA* PCR for confirming resistance to oxacillin.

### Multilocus sequence typing

Seven housekeeping genes (i.e. carbamatekinase (*ara*C), shikimate dehydrogenase (*are*E), glycerol kinase (*glp*), guanylate kinase (*gmk*), phosphate acetyltransferase (*pta*), triosephosphateisomerase (*tpi*), and acetyl coenzyme A acetyltransferase (*yqiL*)) were sequenced to determine the MLST type of each isolate. The sequence results were compared with the sequences in the *S*. *aureus* MLST database (http://saureus.mlst.net/). The MLST type was determined by the corresponding allelic numbers of the 7 genes [23].

### MALDI-TOF MS spectra and data processing

MALDI-TOF spectra were used to identify the species of all isolates, as described in the preceding paragraph. The measurement procedures were conducted following the manufacturer’s instructions (Bruker Daltonik GmbH, Bremen, Germany). In brief, the isolates were subcultured from milk stock onto trypticase soy agar containing 5% sheep blood (Becton Dickinson, MD, USA) at 37°C for 22–24 h. Single colony was scraped from agar plate onto MALDI-TOF steel target plate. The microbial film was then overlaid with 1 μl 70% formic acid. After air dried in room temperature, one micro-litter matrix solution (50% acetonitrile containing 1% α-cyano-4-hydroxycinnamic acid and 2.5% trifluoroacetic acid) was applied. Again after air dried, MALDI-TOF spectra were conducted on Microflex LT mass spectrometer (Bruker Daltonik GmbH, Bremen, Germany). Spectra were obtained under linear positive mode with +20 kV as the accelerating voltage, and the nitrogen laser frequency was set as 60 Hz. Two hundred and forty laser shots were collected from different locations over each sample spot. All samples were assayed in duplicate. Bruker Daltonics Bacterial Test Standard was used for external calibration for the spectra. Flexanalysis 3.4 (Bruker Daltonik GmbH, Bremen, Germany) was used for mass spectra processing, which was set as following: Savitzky-Golay algorithm for smoothing; Top Hat method for baseline subtraction; relative intensity threshold was set as zero; signal-to-noise ratio threshold was set as two. The species of *S*. *aureus* was reported on the basis of analysis results from Biotyper 3.1 (Bruker Daltonik GmbH, Bremen, Germany). Biotyper provided peaks with sufficient intensity and signal quality. For each isolate, the number of peaks was set up to 100. MALDI-TOF analysis of isolates was repeated until an acceptable quality level of spectra (log score ≥ 2.00) was obtained, in accordance with the benchmark instruction of Biotyper 3.1. In this investigation, spectra ranging from 0 to 20,000 Da were obtained for further analysis.

### Analysis of MS spectra

A type template was generated for each major MLST type according to the m/z value. In brief, the m/z values of peaks were aligned among the isolates of an identical ST type. If a particular m/z value had a high probability of occurrence for a particular ST type, it was selected as a peak feature in the “type template” for the ST type. A low probability such as 20% could be selected as the threshold for preliminarily selecting peak features without too much missing. The signals with highest occurrence probability in local region (±5 m/z) would serve as the temporary centers of the peak features. The locations and variance of the peak features were calculated on the basis of the signals in the local regions. The mass spectrum of each isolate was then aligned against the type templates of the major ST types, resulting in “matched vectors” of log-transformed intensities. The criteria of spectra alignment were based on the location and the location variance of each peak feature. If a peak of mass spectrum was located within mean ± 1.96-fold standard deviation of a specific peak feature location, then it means that there is signal over the specific peak feature. A log-transformed intensity would be put into the corresponding site of matched vector. The matched vectors were then concatenated into a single “integrated vector,” in which each element is the log intensity value of a peak corresponding to a representative peak feature in the type templates. The integrated vectors generated from all isolates were then used to construct ML models for strain type discrimination. The details are illustrated in S1 Fig.

### Illustration of discriminative features

In this study, the DT was applied for illustration of discriminative features. The DT is a simple but widely used ML method, and it was originally intended for resolving classification problems [24]. The DT constructs a flowchart-like decision tree structure in a top–down manner from the training data of a given classification problem, where each internal node denotes a test on a feature, each branch denotes an outcome of the test, and each leaf node represents a class label. At each internal node, the best feature is chosen to partition the data of this node into individual classes. The features that appear in the tree are assumed to be relevant to the given classification problem. In this study, the tree structure was constructed using J48 in WEKA [25]. The integrated vectors were normalized such that each vector element was 0 or 1. A decision tree was built from the normalized vectors by using the DT in order to identify the most feasible features relevant to the discrimination of MRSA strain types. The features at the internal nodes of the tree structure were selected as informative features for the preliminary profile analyses in the following experiments.

### Profile analysis of the discriminative features

For peak profile analysis, radar chart construction was performed to preliminarily investigate the differences among the studied ST types. On the basis of the features selected by DT, the mean log intensities of the features were calculated and compared among various ST types. The comparison was illustrated by constructing a radar chart.

### Principal component analysis

Principal component analysis (PCA) was conducted on the selected features to illustrate the distribution of various ST type isolates. The log intensities of the selected features were projected onto principal component 1 (PC 1) and principal component 2 (PC 2). Through the new axes of PC 1 and PC 2, variances among different ST types were maximized, and separation of the various ST types was illustrated. PCA was performed using the “princomp” function of MATLAB (MathWorks, MA, USA).

### Feature selection and construction of predictive models

S2 Fig depicted the framework of training and validating ML models. Nested cross validation was used for the validation [26, 27]. Briefly, we used five-fold cross validation for training (four folds) and validating (one fold) the ML models. Feature selection was performed in the training set of each five-fold iteration by using FSelector (0.21) package of R software (version 3.3.2, R Foundation for Statistical Computing, http://www.r-project.org/). Features were selected using random forest algorithm. Most discriminative features would be selected based on its relevance to the outcome. The selected features were then used for training. Another inner five-fold cross validation was used for tuning ML models within each training set. Feature selection and tuning ML models were performed within training set of each iteration (i.e. nested cross validation) to avoid over-fitting.

### Development of decision tree (DT) models

The classification of a query sample of an unknown class is a top–down process that tests the feature values of the sample against the nodes of the decision tree. It starts from the test of the root node and follows the appropriate branch based on the test. If another node is reached, the test of the node is applied subsequently. If a leaf is reached, the class label associated with the leaf is assigned to the query sample and the classification process is terminated. DT discrimination models based on both binary and multiclass classification strategies were generated using the “ClassificationTree.fit” function of MATLAB (MathWorks). The minimal number at each leaf was set at 2, “deviance” was set for the parameter of “SplitCriterion,” and other parameters were set as default, unless specified. The likelihood of a query sample belonging to a class was estimated using the “predict” function of MATLAB, and the sample was assigned to the class with the highest likelihood.

### Development of support vector machine (SVM) models

In this study, SVM models were constructed using a MATLAB version of the LIBSVM 3.20 software package, which is a widely applied SVM software tool [28]. An effective SVM model was constructed using the procedure outlined in the manual [29]. Briefly, the procedure mainly included two steps: (1) selecting an adequate feature mapping kernel function to map the training samples of two classes into a high-dimensional space such that the two classes might become linearly separable and (2) determining parameters *c* (the penalty for misclassification) and γ (the standard deviation of the radial basis function [RBF] kernel). As described in a number of previous works [30–34], the RBF is a reasonably best choice for a kernel function when training an SVM classifier. The RBF function is defined as K(*S*_*i*_, *S*_*j*_) = exp(−*r*‖*S*_*i*_ − *S*_*j*_‖^2^). Two supporting parameters, gamma (r) and cost (c), are used to enhance the predictive power of the SVM. The RBF kernel is determined by the gamma parameter, while the cost parameter controls the hyperplane softness. A Python program (grid.py) provided by LIBSVM was used to optimize gamma and cost and to obtain a better predictive accuracy. The advantage of the RBF kernel was confirmed in our preliminary trial. Subsequently, another 5-fold cross validation was used for tuning the best values of *c* and γ in each five-fold cross validation, as described previously [18, 35]. Both binary classification and multiclass classification were applied separately to generate the classification models.

### Development of *K*-nearest neighbors (KNN) models

The proposed KNN models were constructed using the “ClassficationKNN.fit” function of MATLAB. Binary and multiclass classification strategies were used to generate the KNN models separately. Another 5-fold cross validation was used for tuning the number of the nearest number (k) in each 5-fold cross validation. Moreover, “cosine” was set for the parameter of distance in the “ClassficationKNN.fit” function. Other parameters were set to their default values. In the KNN models, a case was classified by a majority vote of its neighbors. For each validation case in the validation data set, the distances from the cases in the training data set were calculated. The class categories of the k cases whose distances were closest to the validation case were recorded. The class of the validation case was accordingly predicted based on the major class category of these k closest cases.

### Validation and comparison of various predictive models

The receiver operating characteristic (ROC) curve was used to evaluate the performance levels of the ST type binary classification models based on the DT, SVM, and KNN. ROC curves were generated using SPSS (Version 20; SPSS Inc.). Furthermore, discrimination accuracy and the area under the curve (AUC) were calculated to compare the discrimination abilities of the models. The performance levels of multiclass classification models were also examined by five-fold cross validation. The samples were shuffled randomly before each cross-validation procedure.

### Statistical analyses

To evaluate the performance levels of the ML models, one-way analysis of variance (ANOVA) with a statistical significance level of 0.05 was used to examine the effect of different ML methods (DT, SVM, and DT) on the AUC and discrimination accuracy. The Tukey honestly significant difference (HSD) post hoc test was used to determine the differences when the null hypothesis of ANOVA was rejected. *P* values less than .05 and .01 were labeled separately. All statistical analyses were performed using SPSS (Version 20; SPSS Inc.).

## Results

### Data statistics of eligibly spectra peaks through Biotyper

For the 125 MRSA isolates, the identification scores of *S*. *aureus* provided by Biotyper were all more than 2. The 125 isolates were categorized into three major ST types (ST5, ST59, and ST239) and one minor ST type (ST45). The ST types of the isolates were as follows: 28 isolates were ST5, 8 were ST45, 27 were ST59, and 62 were ST239. The number of peaks ranging from 0 to 20,000 Da was 93.96 ± 12.90 on average. Specifically, it was 91.68 ± 18.44 for ST5, 89.38 ± 20.06 for ST45, 94.22 ± 11.51 for ST59, and 95.47 ± 8.81 for ST239. Moreover, 89 representative peak features were noted in the type template for ST5, 108 for ST59, and 101 for ST239. No type template was built for the minor ST type ST45 because the number of ST45 isolates was not large enough and they were not considered sufficiently representative consequently. The spectral peaks of each isolate were aligned with the 298 (89 + 108 + 101) representative peak features to generate an integrated vector for each isolate.

### Illustration of discriminative features

**Fig 1:**
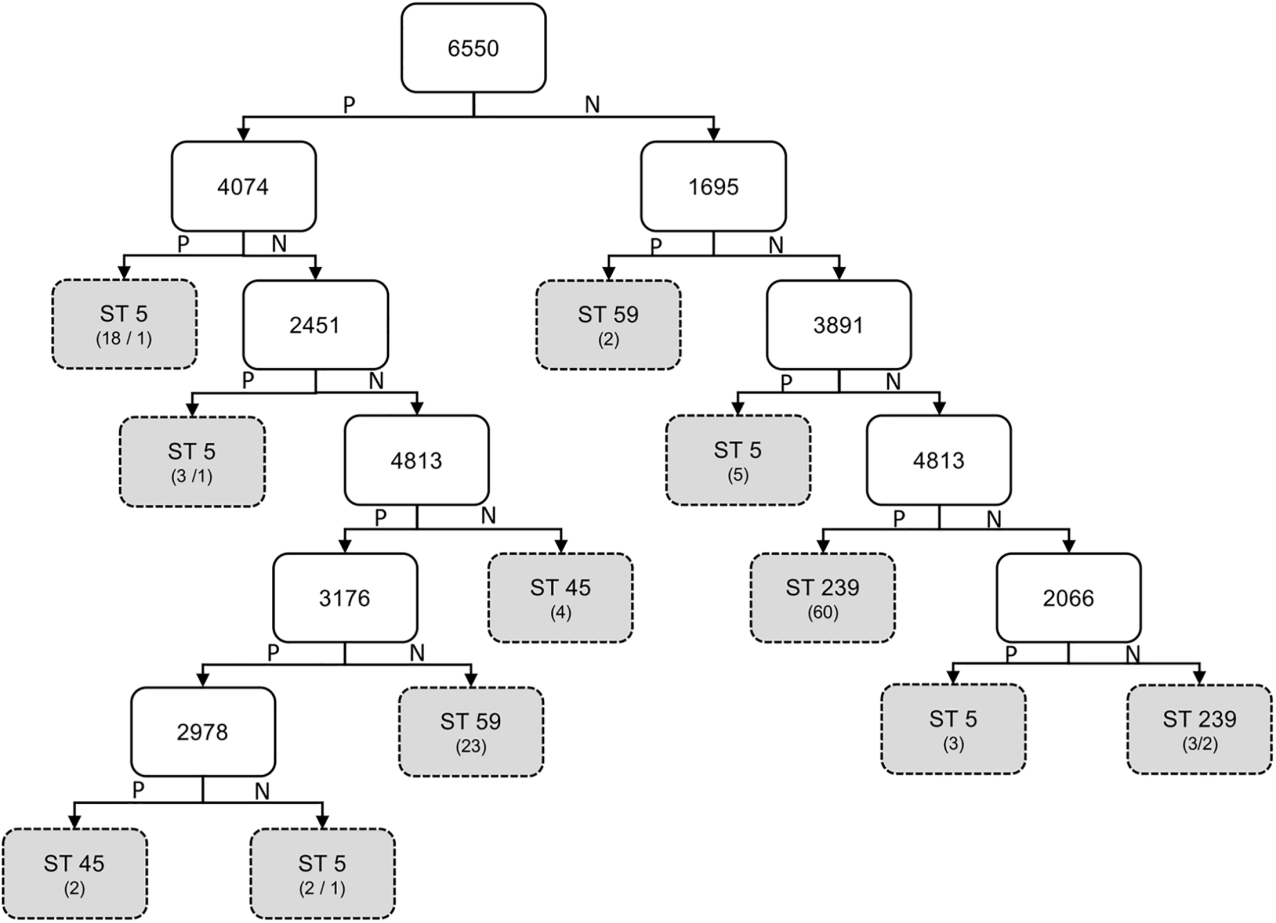
Decision tree structure constructed for illustration of discriminative features. “P” represents “positive” (presence of a peak) at branches of internal nodes; “N” represents “negative” (absence of a peak) at branches of internal nodes.

The DT was used to generate a decision tree structure to select the most discriminative features from the 298 representative peak features. The decision tree comprised 10 internal nodes and 11 leaf nodes, where each internal node denotes a test on a peak feature, and each leaf node represents a class label (ST type). Totally, nine different peak features (m/z values: 1695, 2066, 2451, 2978, 3176, 3891, 4074, 4813, and 6550) were extracted from the decision tree structure, as illustrated in Fig 1.

### Peak characteristics of various ST types

**Fig 2:**
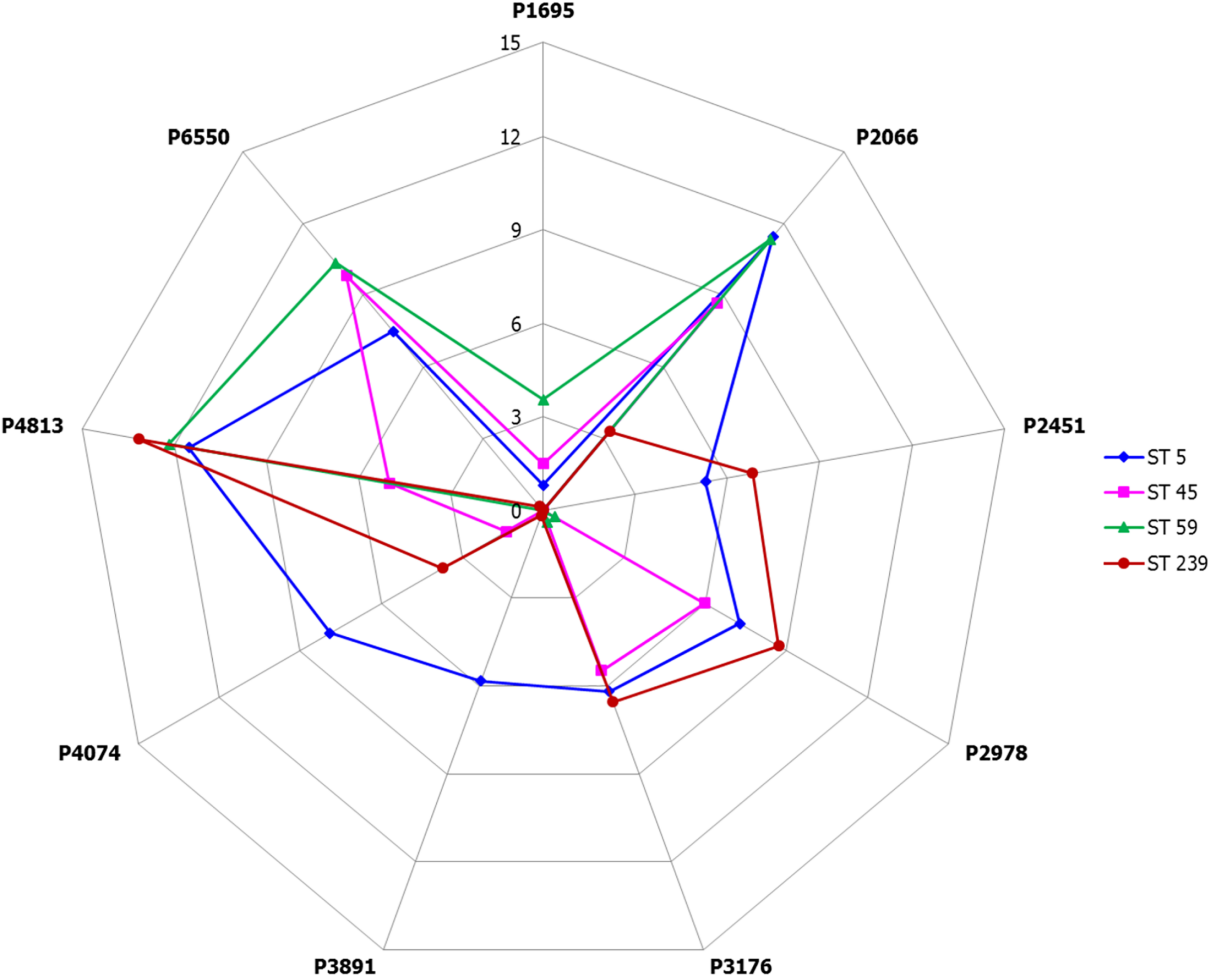
Radar chart of average log-transformed intensities of selected feature peaks. P1695, P2066, P2451, P2978, P3176, P3891, P4074, P4813, and P6550 represent the signal peaks at 1695, 2066, 2451, 2978, 3176, 3891, 4074, 4813, and 6550 m/z, respectively.

Fig 2 presented the radar chart of average log-transformed intensities of the nine selected peak features among the different ST types. Differential peak profile patterns were obtained for the various ST types using the nine features. Comparatively, ST5 showed significantly higher peak intensity than the other ST types at 3891 and 4074 m/z; ST45 showed significantly lower peak intensity at 4813 m/z; ST59 showed significantly higher peak intensity at 1695 m/z and significantly lower peak intensity at 2978 and 3176 m/z; and ST239 showed significantly lower peak intensity at 6550 m/z and higher peak intensity at 2451 and 2978 m/z.

### Three-dimensional scatter plot and PCA comparison of various ST types

**Fig 3:**
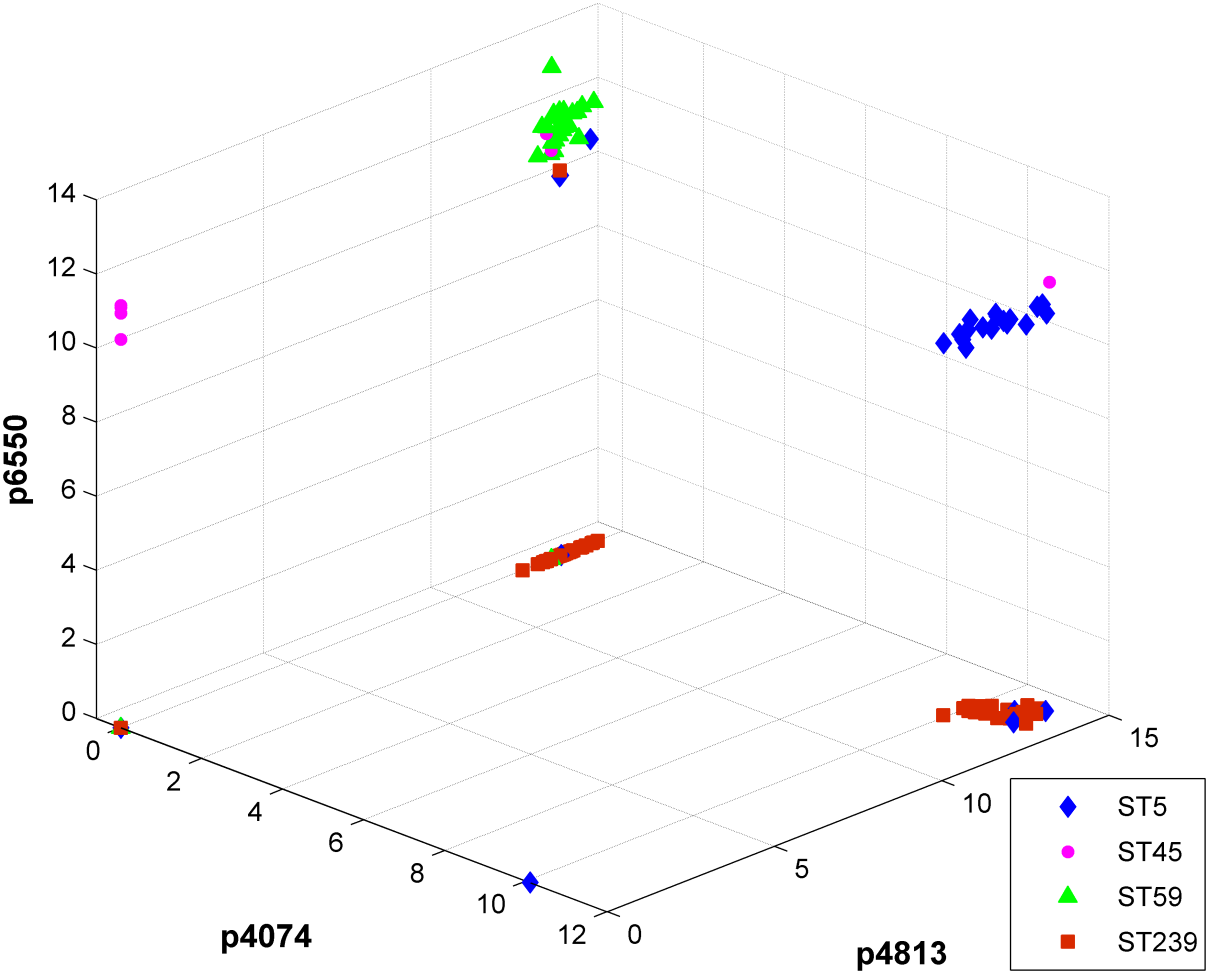
Three-dimensional scatter plot of various ST type isolates. P6550, P4074, and P4813 represent peak signals at 6550, 4074, and 4813 m/z, respectively.

**Fig 4:**
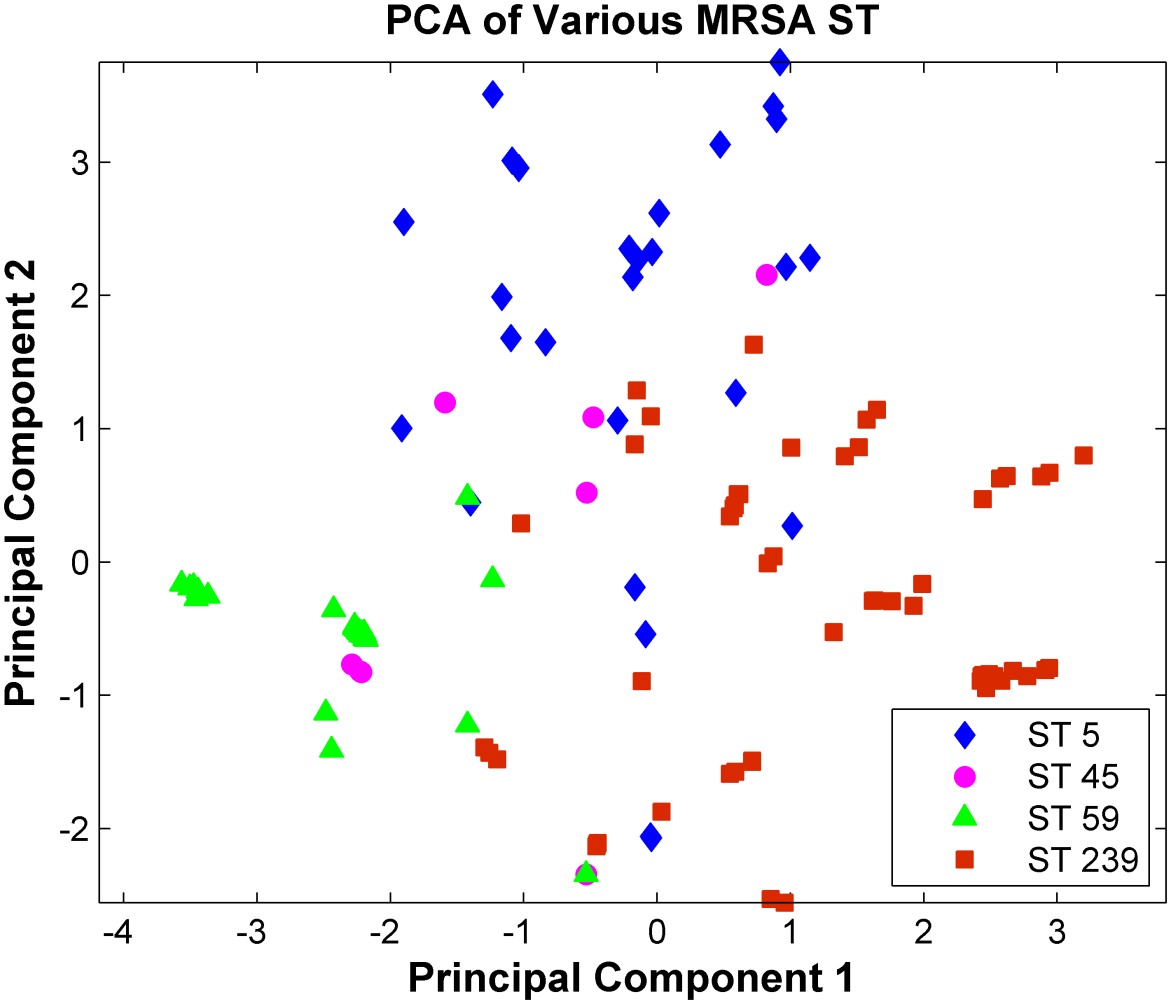
PCA comparison of various ST type isolates.

Three discriminatory peaks (peaks at 4074, 4813, and 6550 m/z) were selected from the nine peak features in the constructed decision tree structure (Fig 1) as axes for the 3D scatter plot. The isolates of various ST types were presented by a 3D visualization (Fig 3) according to the log-transformed intensities of the three peaks. The scatter plot preliminarily revealed that ST5, ST59, and ST239 could be discriminated with relatively high accuracy by using the log intensities of the three selected peaks. Moreover, the isolates of various ST types were plotted on the PCA plot according to the scores over PC 1 and PC 2 (Fig 4). The plot illustrated that ST59 isolates were clustered in a certain area, which could be distinguished with high accuracy from the other clusters. By contrast, the clusters of ST5, ST45, and ST239 could not be clearly separated from each other on the basis of the PCA plot.

### Performance comparison of various ML methods

In this investigation, AUC, and accuracy were used to assess the performance levels of the various ML models for strain type discrimination. For each ST type, the performance levels of the different ML methods based on binary classification were compared (Table 1). For the discrimination of all ST types, the SVM and KNN methods outperformed the DT (*P-value* < 0.01). Moreover, the SVM method generated higher performance than the KNN method for all ST types. In multiclass classification, the performance levels of the various ML models were also compared. The results revealed that all ML methods provided similar performance, with accuracy levels of more than 83% (Table 2).

**Table 1:** AUC of binary classification ML models for different ST types.

ML models	ST5		ST45		ST59		ST239	
	AUC	SE	AUC	SE	AUC	SE	AUC	SE
**DT**	0.762	0.079	0.500	0.000	0.933	0.021	0.895	0.019
**SVM**	0.963	0.018	0.919	0.039	0.975	0.015	0.991	0.005
**KNN**	0.953	0.033	0.789	0.114	0.958	0.026	0.967	0.020

AUC: area under ROC curve; SE: standard error.

**Table 2:** Accuracy of multiclass classification using various ML methods.

ML Methods	ACC	SE
**DT**	0.832	0.015
**SVM**	0.864	0.020
**KNN**	0.848	0.027

ACC: accuracy; SE: standard error.

## Discussions

In this investigation, the ML models could provide a rapid identification of invasive clones, such as MRSA ST239 [36]. Moreover, the models may facilitate the identification of an outbreak of less common strains (e.g., ST45) in a less computationally intensive manner. For binary classification of ST typing, the SVM models outperformed the DT or KNN models (Table 1). Moreover, the KNN models outperformed the DT in the typing of all ST types investigated in this study. By contrast, the DT exhibited low performance for the typing of ST45. In this study, only eight isolates of ST45 were detected. According to our results, ST45 is not a relatively typical epidemiological strain in Northern Taiwan. The ratio of ST45 isolates to non-ST45 isolates was 8:117. The unbalanced data would lead to unfavorable AUC performance of the DT. In this study, multiclass classification models were also developed. All ML models exhibited high performance, with accuracy levels of approximately 85%, and no statistical differences were observed in their accuracy levels (Table 2). However, the performance levels of multiclass classification models are generally not as high as those of binary classification models. A binary classification problem involves only two classes, whereas a multiclass classification problem involves multiple classes. The increased complexity in the number of classes results in the subsequent decline in the classification accuracy.

MALDI-TOF mass spectra could be used to facilitate the characterization of multiple biological molecules, mainly proteins. For typing at the species level, the expression patterns of certain proteins, particularly ribosomal proteins, are used as fingerprints of specific species [9, 13]. Recently, identification of genus and species are with high accuracy in clinical microbiology laboratories. However, for highly related isolates of the same species, subspecies typing by using MALDI-TOF spectra has not been well-established yet. MALDI-TOF mass spectra are composed of multiple signals of peaks, including m/z values, intensities, areas, and signal-to-noise ratios. Manual analysis of the massive and complex omics data is labor intensive. Recently, visual examination has been adapted to identify the representative peaks on pseudo-gel spectra. The accuracy of the method depends on the experienced personnel, and this method is relatively labor intensive. The characteristics of subjectivity, operator dependence, and limited throughput restrict the application of the method. In addition, compared with MALDI-TOF mass spectra among different species, more subtle differences are observed in the spectra among different strains [15, 37]. Consequently, it is reasonable to incorporate ML methods to provide a more unbiased analysis. For successfully applying ML methods, data preprocessing is a crucial step. To identify the relevant peak features of MALDI-TOF mass spectra for strain typing, a type template was established for each ST type in this study. The approach resolved the problem of peak shift [22] and facilitated feature definition, which is important for ML-based analysis. The features in the type template of a specific ST type denote characteristic m/z values that can serve as a fingerprint for that ST type. The spectra of each isolate were aligned with the type templates of ST5, ST59, and ST239. Log-transformed intensities at the specific m/z values of the ST type templates were integrated into an integrated vector (as presented in S1 Fig). Through these procedures and alignment, the complex MALDI-TOF spectra of the 125 MRSA isolates were standardized for further analysis.

For classification problems with a large set of features, another essential issue is the selection of a subset of relevant features. Several feature selection methods, including filter, wrapper, and embedded methods, have been developed to avoid the curse of dimensionality for high-dimensional classification problems [38]. Wrapper methods are the most likely to identify the most robust combination of features when exhaustive searching is used to evaluate every combination [38]. However, in this study, 2^298^ − 1 different combinations of features would require significant computation time if a wrapper method was adopted to select a subset of relevant features for developing ML models for strain typing. Thus, using a wrapper method would be computationally intensive and impractical. By contrast, feature importance from random forest algorithm is a simple but effective method for feature selection. In this study, the features selected by random forest could generate high-performance ML models for MRSA strain typing.

The DT, SVM, and KNN approaches are all widely applied ML methods. For classification problems, the DT constructs a decision tree structure to extract simple *if-then* classification rules that enable intuitive interpretation and explanation of the classification results. By contrast, the classification rules in the SVM and KNN methods are implicit and cannot be explained easily. For the SVM method in this study, the samples of two different classes were mapped into a space of higher dimension by an RBF kernel to enable linear separation between the two classes. The SVM method is also one of the state-of-the art ML methods, which is widely applied in many fields including biomedicine research [18, 39]. By contrast, the KNN approach is an instance-based method. The classification of a query sample is determined on the basis of the instances in the storage database. The KNN method is similar to decision-making in clinical practice, where previous experiences and data are essential. Moreover, it also resembles the identification of bacterial species by Biotyper, which a bacteria database is necessary for the identification.

It was reported that the difference among the MALDI-TOF spectra of bacteria is subtle at the subspecies level [15, 37]. To learn from spectra, information from more than single peaks should be collected [6, 13, 15, 37, 40]. For example, the intensity data of peaks provide information about the protein expression level of *S*. *aureus*. Data including m/z values and their corresponding intensity generate more accurate ST typing models. As illustrated in Fig 3, the intensity differences for peaks at 4074, 4813, and 6550 m/z provided more clues in differentiating various ST types. To discriminate various ST types, the effects of multiple features on classification were investigated. Fig 3 has presented that the isolates of various ST types, except ST45, could be discriminated with relatively high accuracy according to the intensities of peaks at 4074, 4813, and 6550 m/z. When all nine features from the DT structure were selected in PCA (Fig 4), similar results were attained. These investigations have preliminarily demonstrated that the various ST types of MRSA are separable. ML methods were introduced to facilitate higher and systemic classification performance.

Several limitations should be acknowledged. The composition of the microbial strain may predefine the performance of subspecies classification. The performance of strain typing may be possibly compromised due to a complicated composition. In this study, we aimed to develop a framework of applying machine learning method in analysis of MALDI-TOF spectra. We used MRSA for demonstrating the performance of the proposed methodology. The essential feature identified in this study may be not generalized in other area or country. By contrast, a different laboratory may be able to construct a useful typing model by using the proposed methodology to analyze their locally relevant data. To address the issue of microbial strain composition, we also performed spa typing and *SCCmec* typing for each strain. The result revealed a diverse distribution of the microbial strain over ST types, spa types & *SCCmec* types (S3 Fig). Specifically, ST45 & ST59 strains revealed a relative trend of diverse composition, while ST5 & ST239 showed a relative trend of clustering. For the ST59 classifiers, however, the performances were not much compromised than those of ST5 or ST239 (Table 1). Besides, the reproducibility of spectra is also a crucial issue when applying MALDI-TOF spectra for bacterial typing [3, 5, 6, 9]. High inter-laboratory reproducibility has been reported for identification at the genus or species level. For typing at the strain level, reproducibility is even more crucial. The variance between batches should be limited to less than the spectral difference between strains. Various factors affect the reproducibility of spectra, including the specimen type, sample processing, growth stage, culture media, culture condition, and sample deposition on the target plate [1, 6, 9, 13, 40]. High fidelity of spectra can be assured only when all of these factors are well qualified. However, no standard protocol has been proposed for strain typing by using MALDI-TOF spectra. Moreover, the standard protocol may be optimized and specified for each species to obtain robust strain typing performance [5, 6, 9, 40]. In our laboratory, the College of American Pathologists proficiency test has been conducted for years to accredit the performance of all personnel and tests. Thus, on this basis, DT, SVM, and KNN ML models were developed to optimize the interpretation of MALDI-TOF mass spectra for MRSA strain typing. Generally, the binary classification ML models exhibited a high performance level, particularly the SVM models. The accuracy of multiclass classification ML models was approximately 85%. For binary or multiclass classification, the SVM method was generally the most robust ML method in this study (Tables 1 and 2).

## Conclusion

The analysis of MALDI-TOF spectra by using various ML methods exhibited high performance for MRSA strain typing. A presumptive strain typing result could be provided a couple of days earlier before confirming the test results. Subsequently, this strain typing result may benefit infection control or epidemiological investigation in near real time. The promising features include rapidity, cost-effectiveness, objectiveness, and accuracy. On the basis of our results, a standardized protocol should be developed to optimize ML-based microorganism strain typing.

## Supporting information

S1 Fig (a) Detail processes of generating the type templates; (b) Detail processes of using the type templates to generate an integrated vector. (DOCX)

S2 Fig The framework of ML models training and validation. (DOCX)

S3 Fig Diversity of the isolates. (DOCX)
