# Supplementary material for: A new scheme for strain typing of methicillin-resistant Staphylococcus aureus on the basis of matrix-assisted laser desorption ionization time-of-flight mass spectrometry by using machine learning approach
Source: PLoS One. 2018 Mar 13;13(3):e0194289. doi: 10.1371/journal.pone.0194289 (PMC5849341; doi:10.1371/journal.pone.0194289)

**S1 Fig (a).** **Detail processes of generating the type templates**. The depiction illustrated an example of generating a peak feature of type templates. First, the occurrence probability of signals over 3889 m/z is larger than the threshold (20%). 3889 is set as a temporary center. Subsequently, the signals in the local region (±5 m/z) are calculated to determine the location & standard deviation of the peak feature.

**
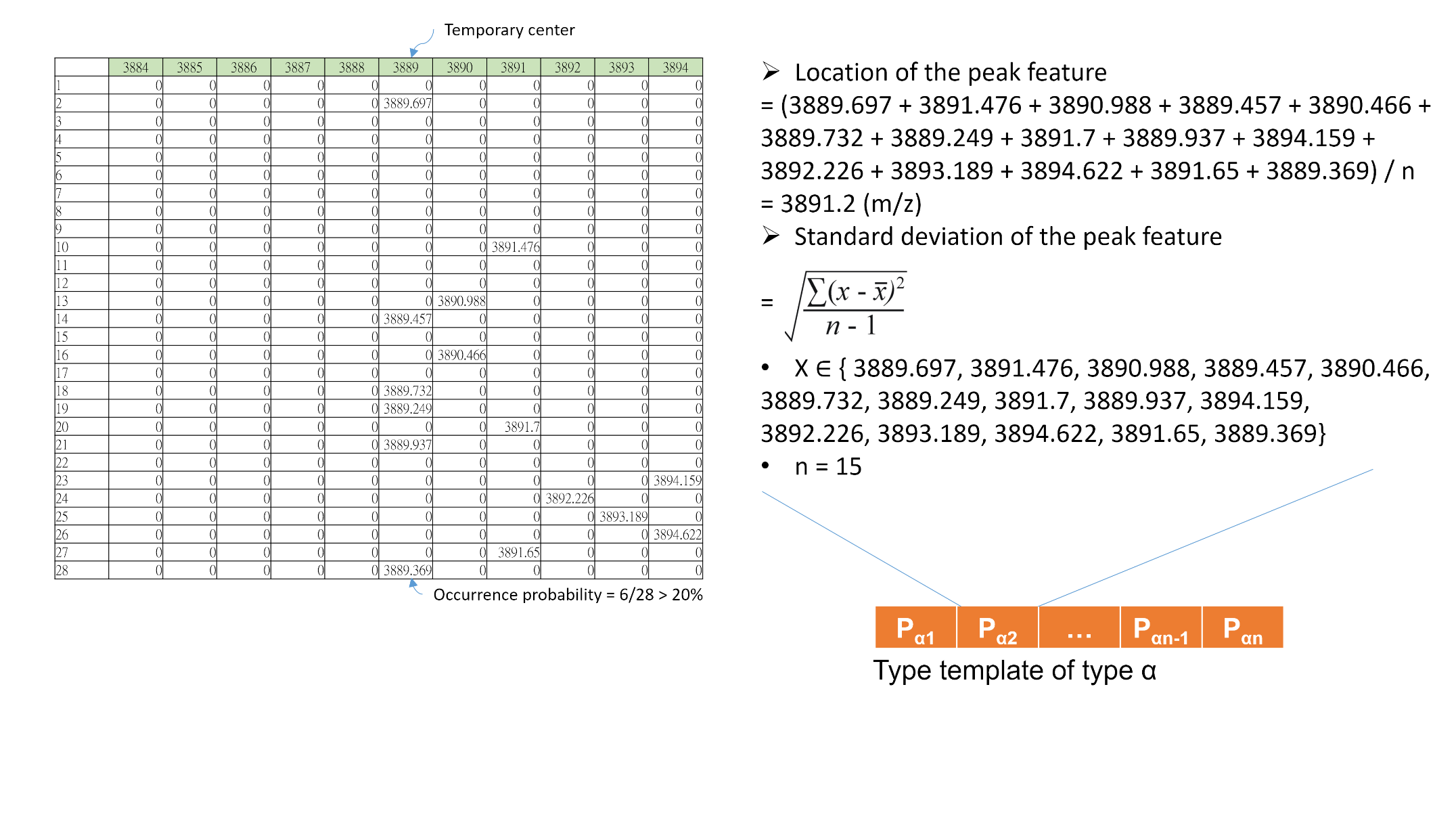
**

**S1 Fig (b).** **Detail processes of using the type templates to generate an integrated vector.** (I) MALDI-TOF spectrum was measured and matched against various type templates (e.g. in the illustration, type templates of type α, β, and γ). Matched vectors were generated (e.g. in the illustration, matched vectors of type α, β, and γ). (II) in the illustration, matched vectors of type α, β, and γ were integrated in order to generate an integrated vector.

(I)


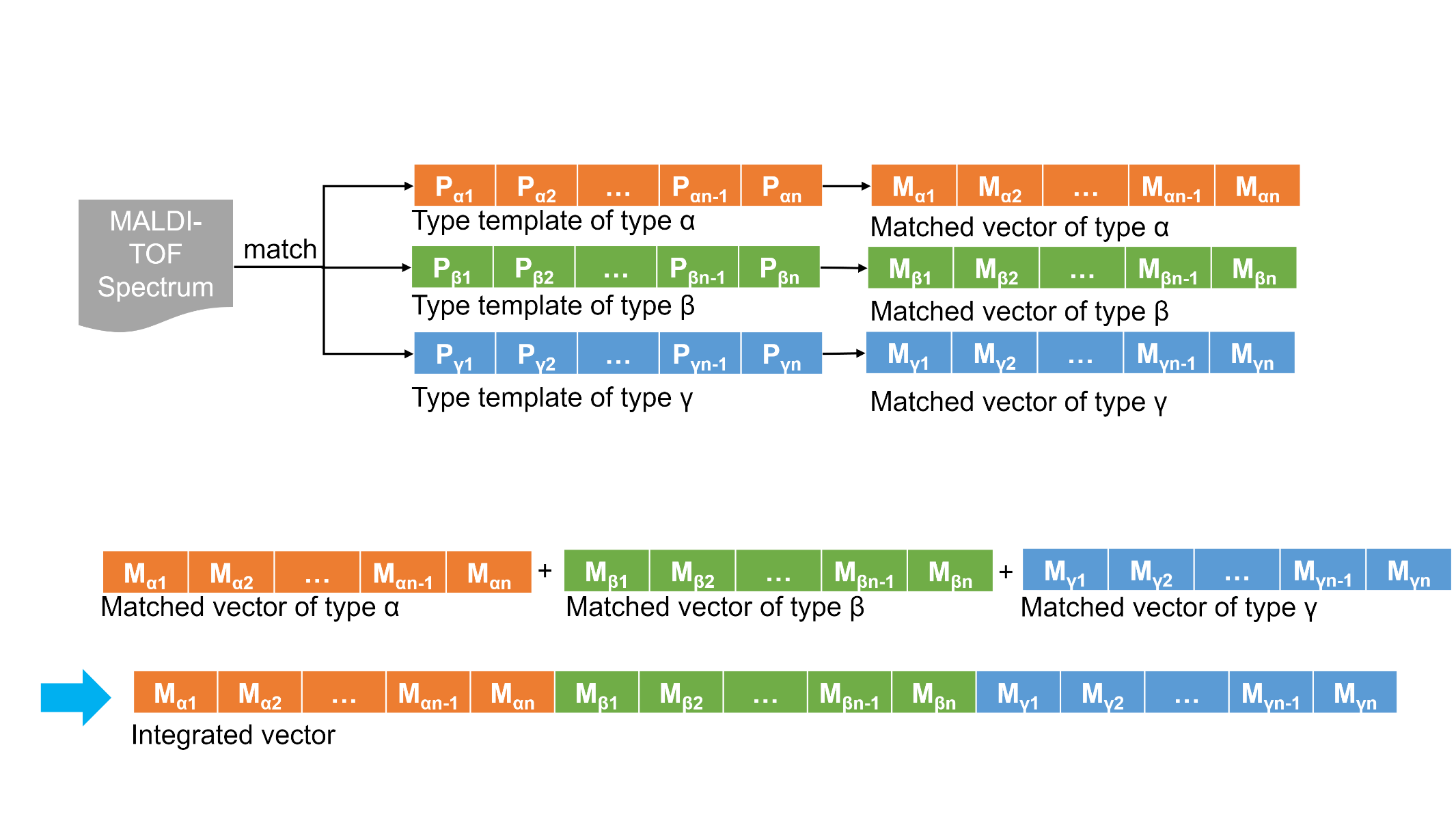


(II)


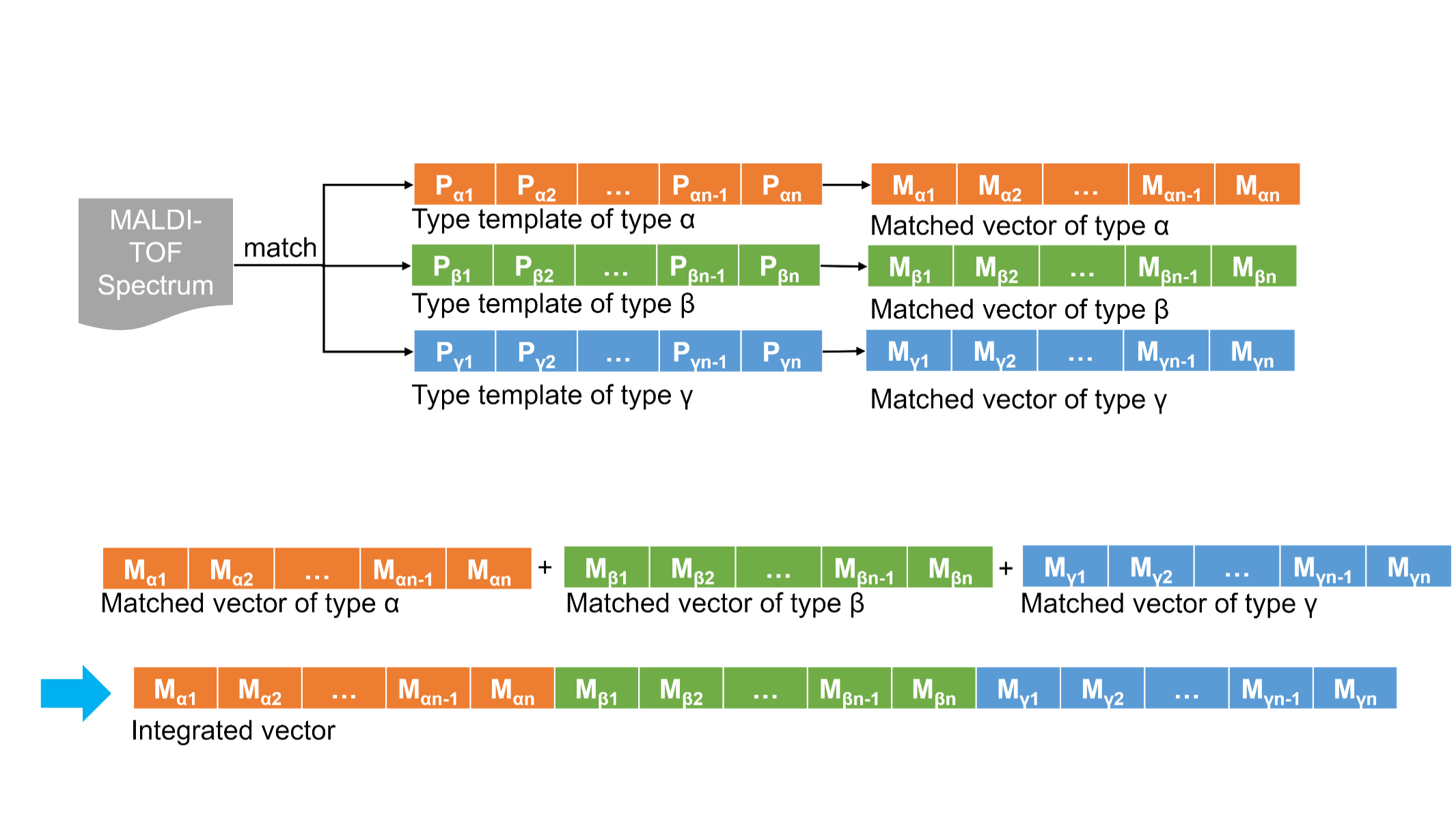

Supplement: S1 Fig — (DOCX) [file pone.0194289.s001.docx]
