# Supplementary figures and images for: A new scheme for strain typing of methicillin-resistant Staphylococcus aureus on the basis of matrix-assisted laser desorption ionization time-of-flight mass spectrometry by using machine learning approach
Source: PLoS One. 2018 Mar 13;13(3):e0194289. doi: 10.1371/journal.pone.0194289 (PMC5849341; doi:10.1371/journal.pone.0194289)

**S2 Fig. The framework of ML models training and validation.** ML: Machine learning.


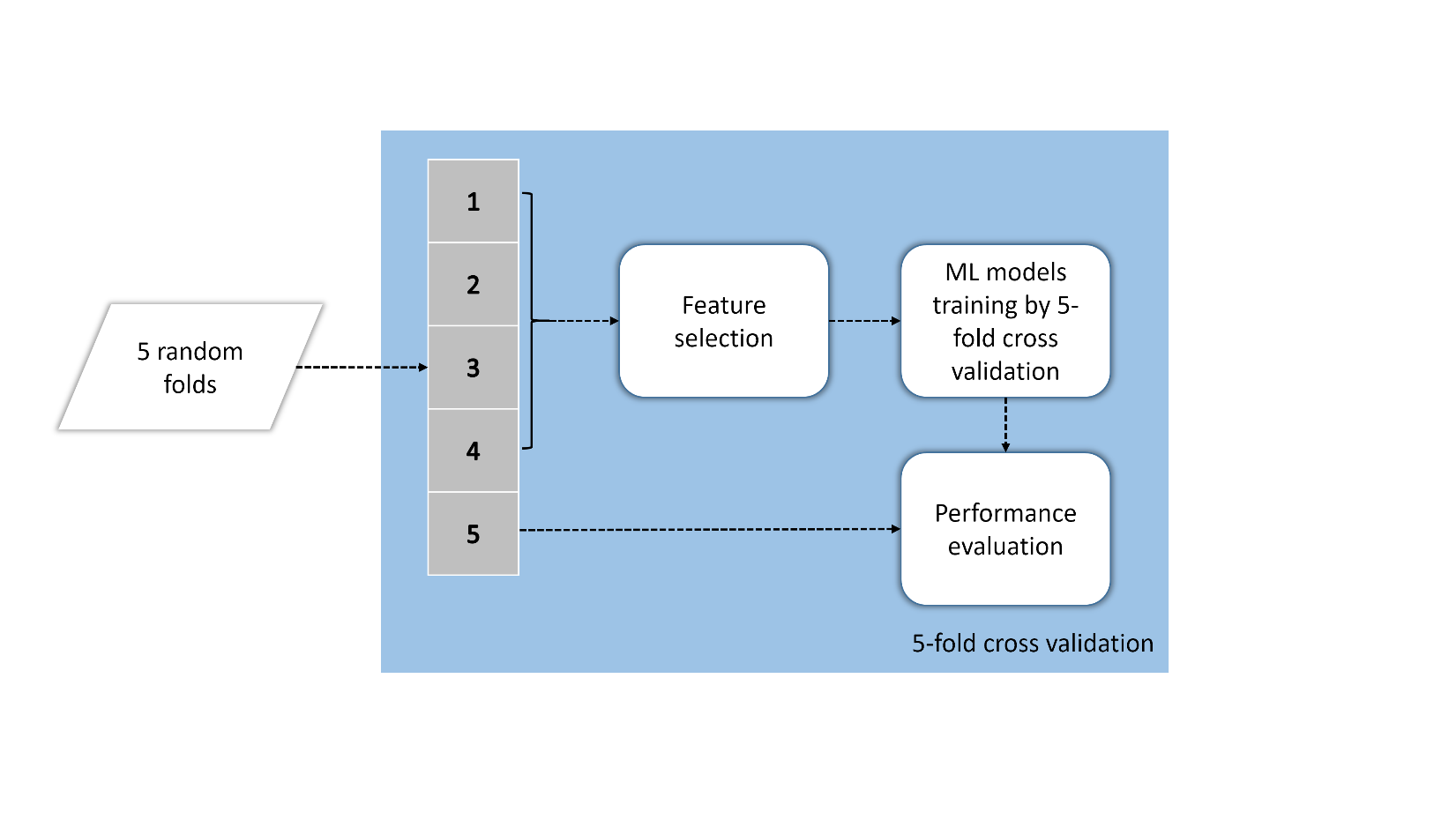

Supplement: S2 Fig — (DOCX) [file pone.0194289.s002.docx]
