# Supplementary material for: A new scheme for strain typing of methicillin-resistant Staphylococcus aureus on the basis of matrix-assisted laser desorption ionization time-of-flight mass spectrometry by using machine learning approach
Source: PLoS One. 2018 Mar 13;13(3):e0194289. doi: 10.1371/journal.pone.0194289 (PMC5849341; doi:10.1371/journal.pone.0194289)

**S3 Fig. Diversity of the isolates.** Three strain typing methods, namely ST typing, SCCmec typing, and spa typing methods were used for demonstrating the composition of the isolated used in this study. Preliminarily, the diversity of composition was relatively higher for ST45 and ST59, while ST5 and ST239 showed a relative trend of clustering.


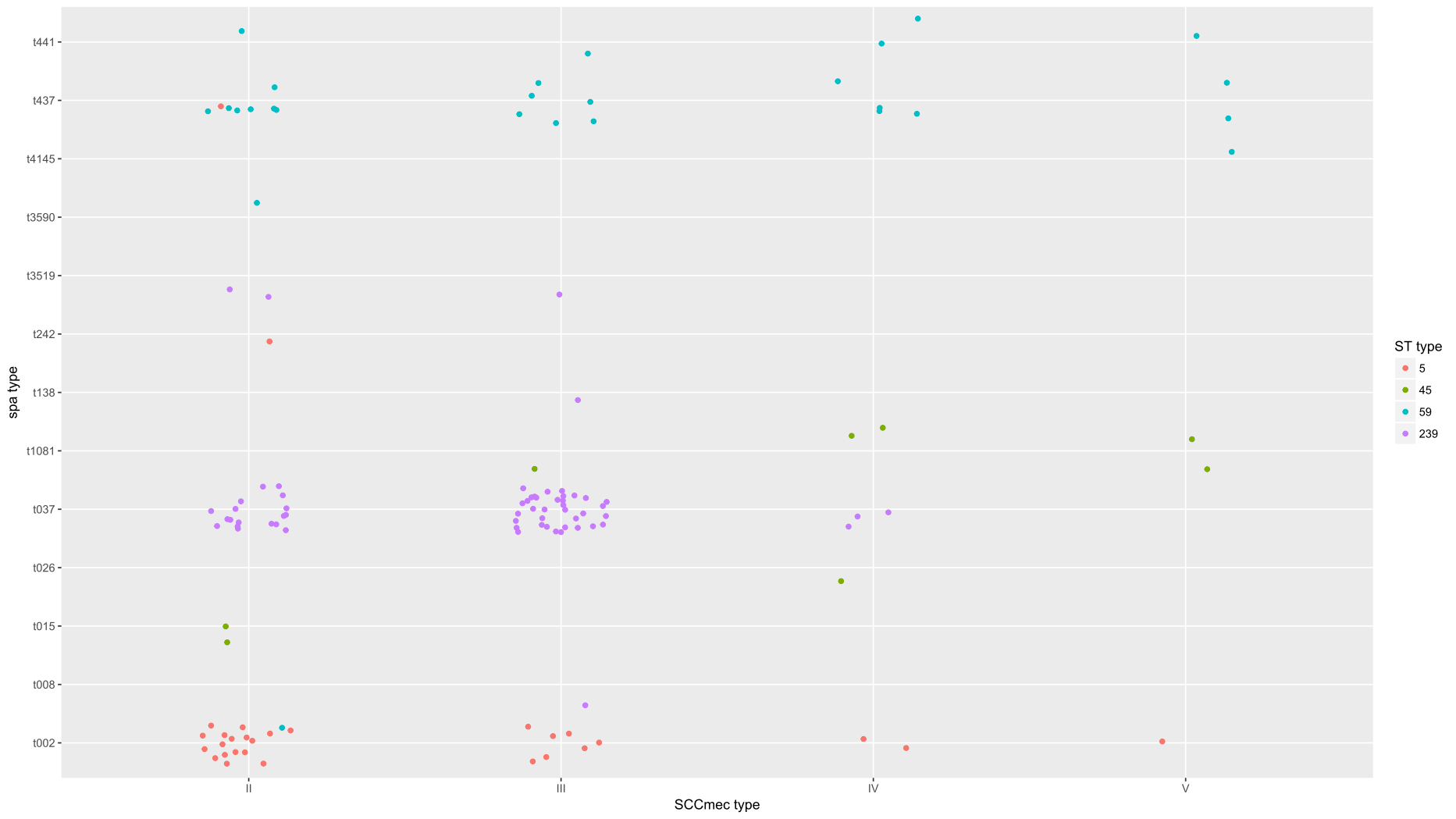

Supplement: S3 Fig — (DOCX) [file pone.0194289.s003.docx]
